# Experiences of Individually Tailored Internet-Based Cognitive Behavioral Therapy During the COVID-19 Pandemic: Qualitative Interview Study

**DOI:** 10.2196/66908

**Published:** 2025-06-11

**Authors:** Victoria Aminoff, Matilda Baltius, Emelie Lundström, Matilda Berg, Gerhard Andersson, Mikael Ludvigsson

**Affiliations:** 1 Department of Behavioural Sciences and Learning Linköping University Linköping Sweden; 2 Department of Biomedical and Clinical Sciences Linköping University Linköping Sweden; 3 Department of Clinical Neuroscience Karolinska Institute Stockholm Sweden; 4 Department of Psychiatry Linköping University Linköping Sweden; 5 Department of Geriatrics and Palliative Medicine, and Department of Health, Medicine and Caring Sciences Linköping University Linköping Sweden

**Keywords:** internet-based cognitive behavioral therapy, COVID-19, internet, thematic analysis, qualitative methods, interview

## Abstract

**Background:**

During the COVID-19 pandemic, both physical and psychological health were at risk. Internet-based cognitive behavioral therapy (ICBT) is a psychological treatment alternative that does not inherently increase the risk of virus transmission because face-to-face interactions are not required. ICBT has been found to be effective for a variety of mental health problems, both before and during the COVID-19 pandemic. Although the experiences of undergoing ICBT have been investigated in previous studies, the specific experiences of participating in ICBT during the COVID-19 pandemic have been less examined.

**Objective:**

This qualitative study aimed to investigate the experiences of participants undergoing individually tailored ICBT with weekly therapist support during the COVID-19 pandemic.

**Methods:**

We approached trial participants who had received ICBT for psychological symptoms related to the COVID-19 pandemic during the summer of 2020. A strategic sample, based on the number of log-ins to the treatment platform, among other factors, was selected in an effort to achieve the highest possible variation. Semistructured telephone interviews were conducted 4 to 6 months after treatment completion, depending on whether the participant was initially assigned to the treatment or control group. Data were transcribed and then analyzed based on thematic analysis.

**Results:**

A total of 16 participants aged between 23 and 78 years were interviewed. Four main themes and 10 subthemes were derived from the thematic analysis: (1) functions of the treatment (initiating and motivating, perspective widening), (2) treatment equals work (experience of the treatment as demanding, going from text to action, posttreatment engagement, participant agency), (3) changes experienced (changes in relation to the COVID-19 pandemic, other changes not related to the COVID-19 pandemic), and (4) wishing for something else (individually tailored, contact with the therapist).

**Conclusions:**

The results closely align with those of previous qualitative studies on experiences of ICBT. Participants expressed appreciation of the treatment’s content and format. Suggestions and wishes for changes were also expressed in the interviews. However, a unique finding was that participants described experiencing changes in well-being related to the COVID-19 pandemic. At the same time, there were also reports of changes in other symptoms not related to the pandemic. Further studies are needed on the experiences of participants who drop out of ICBT and the type of therapist contact they prefer.

## Introduction

### Background

Throughout history, there have been repeated occurrences of infectious diseases [1]. Increased globalization and human mobility, despite advancements in diagnostics and vaccines, have led to more complex consequences of disease spread [1]. The impact extends beyond physical health to include psychological [2], social, political, and economic dimensions [3]. This was salient during previous pandemics [4], as well as during the COVID-19 pandemic. Research investigating psychological health during the COVID-19 pandemic identified a coemerging psychiatric epidemic [5]. In addition to the fear of death it instilled, the pandemic also had an impact on family organization and work routines and led to the closure of public places, companies, and schools [5]. Furthermore, socioeconomic consequences such as unemployment [6] and reduced access to social support because of isolation and quarantine were reported [7]. An increasing number of studies investigating individuals infected by the SARS-CoV-2 virus compared to the noninfected population have found that those who were infected had higher levels of anxiety, depression, and posttraumatic stress symptoms [8,9]. In addition, strategies to prevent the spread of the virus, such as quarantine, were also associated with psychological distress, including symptoms of depression, posttraumatic stress, and insomnia [10,11]. Thus, the COVID-19 pandemic prompted action not only in response to physical health concerns but also in relation to mental health problems [5].

Internet-based cognitive behavioral therapy (ICBT) has been shown in a growing body of research to be effective for depression and anxiety disorders [12] as well as symptoms related to somatic conditions such as tinnitus and chronic pain [13]. ICBT is commonly delivered through a digital platform [14], and although the treatment can be provided with minimal or no therapist support [15], unguided treatments tend to be less effective than ICBT with scheduled therapist support [16]. Therapist-supported ICBT has shown effects comparable to those of face-to-face cognitive behavioral therapy (CBT) [17], with additional evidence supporting its effectiveness and acceptability in routine care settings [18].

An increased number of monthly course registrations for ICBT was observed during the early phase of the COVID-19 pandemic [19]. In line with this, there was a call in the literature for digital psychological health interventions during the pandemic [5,19,20], along with an emphasis on evaluating their effectiveness, quality, and safety [21]. Thereafter, an increasing number of studies have been conducted on ICBT and its effects on psychological symptoms during the pandemic. Our research team conducted a pilot study in the summer of 2020 to explore the effects of individually tailored ICBT during the early phase of the pandemic [22]. The results indicated that the treatment was effective for symptoms of depression, anxiety, and stress in participants who underwent ICBT compared to a waitlist control condition. The findings align with those of Wahlund et al [23], who examined a brief ICBT intervention for dysfunctional worry related to the COVID-19 pandemic. In addition to improvements in worry, the study found reductions in other symptoms, such as depressive symptoms [23]. A few months after the pilot study was conducted by our research team [22], a randomized controlled trial (RCT) of the same treatment demonstrated effects on symptoms of depression and insomnia as well as other reported symptoms [24]. In a meta-analysis, Komariah et al [25] summarized findings from 12 studies conducted during the COVID-19 pandemic and showed that ICBT could decrease symptoms of depression and anxiety. Similar effects of ICBT on symptoms of depression and anxiety have been reported during the COVID-19 pandemic as before the pandemic outbreak [19]. In line with previous research, ICBT with therapist support had superior outcomes compared to self-guided interventions [25], although both therapist-supported and self-guided ICBT were found to have effects on depressive and anxiety symptoms [26]. Most of the ICBT studies examined in the meta-analysis by Komariah et al [25] included participants regardless of whether they had contracted COVID-19. However, Liu et al [27] found improvements in depressive symptoms, anxiety, and insomnia among individuals specifically diagnosed with COVID-19. At the same time, not all studies have demonstrated ICBT to be effective for all aforementioned symptoms; for instance, Brog et al [28] found no effects of ICBT on depressive or anxiety symptoms, although an improvement in emotion regulation skills was observed. Overall, however, ICBT seems to have been a promising treatment option for psychological symptoms during the COVID-19 pandemic, as evaluated quantitatively through self-assessment questionnaires.

Several sociodemographic and psychosocial factors were proposed to be associated with declining mental health during the COVID-19 pandemic. This included, for example, gender—women had a higher risk of developing mental health problems than men [29,30]—and preexisting mental health problems, with comorbid physical and mental health conditions before the pandemic being predictive of worse outcomes [31]. Interviewing participants who underwent ICBT for psychological symptoms during the COVID-19 pandemic could provide a better understanding of how the treatment works for each individual and highlight problems experienced during treatment.

Qualitative methods complement quantitative data by capturing participants’ views, thus providing insights into how interventions are experienced. Several studies have examined ICBT from various research perspectives before the COVID-19 pandemic, investigating participants’ experiences with this form of treatment. These studies have focused on individuals with different mental health conditions who have undergone distinct ICBT programs for, for example, depression [32], anxiety [33], and procrastination [34]. In a systematic review, Patel et al [35] synthesized qualitative research and literature on participants’ experiences of internet interventions aimed at targeting depression, anxiety, and somatoform disorders, including their acceptability and usability. The authors identified 3 main themes (with subordinate subthemes): initial motivations and approaches, personalization of treatment, and the value of receiving personal support [35]. The first main theme concerned the important role played by the participants’ initial motivations and attitudes for engaging with internet interventions; for example, the participants’ approach toward the interventions at the beginning was found to have an impact on both recruitment and the therapeutic process. The second theme emphasized availability and flexibility, which could contribute to an increased sense of autonomy. With regard to the third main theme, Patel et al [35] reported that almost all studies they included in their review had noted the value of human support. The support was acknowledged as helpful in understanding the interventions better and enhancing commitment and motivation [35].

However, fewer studies have examined the experiences of participants undergoing ICBT during the COVID-19 pandemic. Among these, the study by Thapar et al [36] examined both patients’ and therapists’ perceptions of ICBT. The themes identified in the interviews they conducted included facilitators, such as the accessibility of registering for the program and the feasibility of completing the psychotherapeutic content of the program, as well as barriers, such as challenges with the web-based modality for developing a therapeutic alliance and limited opportunities for customization [36]. In another study, Ncheka et al [37] interviewed 50 individuals who had participated in a CBT program called Moodgym [38], identifying themes such as Moodgym improved understanding of how thoughts influence feelings and students experienced some challenges with Moodgym. Within the same field, adolescents’ experiences of dialectical behavior therapy during the COVID-19 pandemic were also investigated, although the treatment was provided via telephone or video calls rather than through text-based modules and asynchronous communication with the therapist [39]. The themes identified included benefits of virtual dialectical behavior therapy and sense of loss [39]. In addition, experiences of CBT via video calls for depression during the pandemic have been explored, focusing on patients’ and therapists’ perspectives [40]. Both similarities, such as clinical efficacy, and differences, such as the structure of therapy, were identified in the experiences of both patients and therapists [40]. Thus, while some studies have focused on participants’ experiences of ICBT during the COVID-19 pandemic, to the best of our knowledge, no study has specifically examined participants’ experiences of individually tailored ICBT during the COVID-19 pandemic. The individually tailored approach could help address the previously noted need for greater ICBT customization [36].

### Objectives

ICBT has been associated with positive experiences both before and during the COVID-19 pandemic. However, some participants have also reported negative experiences or unmet needs within the ICBT programs examined in previous studies. Therefore, further research on ICBT experiences remains necessary, particularly in the context of the COVID-19 pandemic because limited studies have been conducted under these conditions. Thus, this study aimed to investigate and develop a deeper understanding of participants’ experiences of a 7-week individually tailored ICBT program with weekly therapist support during the COVID-19 pandemic, more specifically during the summer of 2020, as well as to investigate the experience of the treatment’s usefulness. The program’s effects were examined separately in a pilot RCT [22].

## Methods

### Study Design

This study was part of a larger RCT project on ICBT [22] (ClinicalTrials.gov NCT04424212). In that RCT project, adult individuals in Sweden with psychological symptoms related to the COVID-19 pandemic were recruited and randomly assigned to either a 7-week ICBT program with weekly therapist support or a waitlist control group. The control group received the same treatment 2 months later, after posttreatment measurements had been collected. The interviews reported in this study were conducted by telephone 4 to 6 months after the treatment ended, depending on whether the participant had been allocated to the immediate treatment or waitlist control group. This manuscript was prepared in accordance with the COREQ (Consolidated Criteria for Reporting Qualitative Research) checklist [41].

### Participants and Recruitment

Participants were recruited for this study from the aforementioned pilot RCT [22]. Briefly, participants were recruited for the RCT through advertisements on social media and in a national newspaper. Participants completed a web-based informed consent form and a pretreatment assessment, followed by a brief clinical telephone interview. As the intervention was provided through a digital platform, and both recruitment and eligibility assessment were conducted via the internet and telephone, individuals from across Sweden could apply to participate. A complete list of the inclusion and exclusion criteria for the pilot RCT is provided in the study by Aminoff et al [22]. The exclusion criteria were, among others, severe mental or somatic illness or acute suicidality that could hinder participation. Individuals meeting these criteria were referred to other health care services and encouraged to seek help through them (health care in Sweden is tax funded and does not require private insurance).

For this study, a strategic sampling approach aimed at maximum variation was used: we included participants from both the immediate and waitlist treatment groups in the pilot RCT [22]. Recruitment began with an email to 23 participants outlining the study’s purpose and informing them that they had the right to decline or withdraw participation at any time without providing a reason and that a research team member would contact them by telephone to schedule an interview. Individuals who had not engaged with the treatment at all and those who had dropped out were excluded because the aim of the study was to explore participants’ experiences of undergoing ICBT during the early phase of the COVID-19 pandemic. During the brief clinical telephone interview conducted after the treatment in the pilot RCT, participants had rated their improvement using the Clinical Global Impressions–Improvement scale [42], which measures perceived change in well-being, comparing the state just after treatment to the state before treatment. Participants rate this change on a 7-point Likert scale, ranging from 1 (very much improved) to 7 (very much worse). We used these ratings to gather a broad range of experiences. Participants with varying numbers of log-ins to the treatment platform were also considered [14] because log-in frequency may reflect different levels of engagement with the treatment. Other aspects considered when striving for maximum variation in the sample were age, gender, educational level, and employment status.

### The Pilot RCT Intervention

The pilot RCT treatment was a 7-week individually tailored ICBT program that included weekly therapist support. All participants received the same first and last module, namely, “Introduction” and “Conclusion and maintenance plan.” For the remaining 5 modules, the participants could indicate their preferences. The final selection was made by the research team based on pretreatment assessments, the clinical telephone interview, and the participants’ stated preferences. The therapists involved in the treatment were 1 licensed clinical psychologist and 3 recently graduated clinical psychologists with experience in ICBT who were supervised by the licensed clinical psychologist. The participants received 1 module per week, chosen from 14 possible modules. These optional modules focused on cognitive techniques, behavioral activation, emotion regulation, acceptance, stress management, perfectionism, relaxation, sleep strategies, problem-solving, anxiety and exposure, anxiety and worry, anxiety and panic, social anxiety, and imaginal exposure for difficult memories. In line with the purpose of the pilot study [22], the treatment modules aimed to address psychological symptoms related to the COVID-19 pandemic. The modules were adapted to the recommendations regarding the COVID-19 pandemic issued by Sweden’s Public Health Agency. A detailed description of the modules is provided in the study by Aminoff et al [22]. All communication between the participants and the therapists took place on the same platform where the modules were delivered [14].

### Data Collection

The interviews were conducted in February 2021 by telephone, allowing both interviewers and participants flexibility regarding location. The interviewers (M Baltius and EL) were either at home or at Linköping University, while the participants were at home. No other person was present with the interviewers, and to the best of our knowledge, no one else was present with the participants, except for one who mentioned holding her infant during the interview.

The interviewers had no contact with the participants before the study. The interviews began with the interviewers introducing themselves and reiterating the purpose and procedures of the study. Informed consent was also obtained at this stage. Pseudonyms have been used in the Results section to preserve the participants’ anonymity. The semistructured interviews were conducted using an interview guide (Multimedia Appendix 1), adapted from Beukes et al [43], which was not pilot-tested because its structure and questions had previously proven adequate. The questions were mostly open ended and exploratory. Participants were asked about their expectations of the treatment as well as their experiences of the treatment and the contact with the therapists with questions such as “How did you experience the treatment?” “How has the treatment affected you and your situation?” Follow-up questions were asked to obtain richer and more detailed data. All interviews were conducted in Swedish, audio recorded, and then transcribed verbatim. The transcripts were not returned to the participants for validation or correction. The recordings were made using the Voice Memos app on an Apple device, transferred to a USB drive, and then deleted from the app. All transcriptions were saved on the same USB drive. The mean interview length was 19 minutes and 11 seconds (SD 7 min and 27 s), with the shortest interview being 8 minutes and 11 seconds and the longest being 35 minutes and 22 seconds.

### Data Analysis

#### Overview

Data were analyzed using thematic analysis (TA) following the guidelines proposed by Braun and Clarke [44]. TA is used to identify and analyze patterns or themes that are meaningful to the research questions [44]. We chose TA with an inductive, bottom-up approach to openly explore and encapsulate data, rather than generate new theory or compare findings to previous research. In contrast to a deductive approach, this method is considered to be more data driven, not aiming to fit the data into already existing frameworks. However, it remains important to acknowledge that researchers’ theoretical and epistemological orientations influence the analysis [44]. To explore the participants’ experiences of the ICBT program, an essentialist and realist approach was adopted.

In line with the analysis procedure described by Braun and Clarke [44], we conducted the qualitative analysis according to the 6 phases outlined in the following subsections. Although Braun and Clarke [44] describe the analysis in 6 phases, they emphasize its recursive nature. Accordingly, we moved back and forth between phases rather than following a strictly linear process.

#### Phase 1: Familiarization With the Data

Initially, we transcribed the interviews verbatim and familiarized ourselves with the data by thoroughly reading the transcripts. An overview of the data material was established by actively and repeatedly reading all transcripts. This process also involved generating keywords and noting preliminary ideas.

#### Phase 2: Generating Initial Codes

We then systematically analyzed the entire dataset and generated initial codes. Codes represent the most fundamental element that can be seen as meaningful regarding the phenomena under study [44]. Thus, the data were organized into relevant categories upon which further analysis could be built.

#### Phase 3: Searching for Themes

Codes are narrower than themes, which we sought in this stage. Themes, defined as elements capturing something relevant to the phenomenon and research question being investigated [44,45], were identified by grouping codes that were assessed as sharing common characteristics. Themes reflect patterns in the data rather than their frequency [44] and were developed based on their capacity to meaningfully represent aspects of participants’ experiences relevant to the research aim.

#### Phase 4: Reviewing Themes

In an iterative process, we sought codes, subthemes, and themes and compared them to each other. This iterative process aimed to digest and synthesize the findings continuously, as well as to enhance the confirmability of the analysis. Following the recommendations by Braun and Clarke [44], this process involved 2 stages: first, reviewing and refining the themes; and second, comparing them to the entire dataset. We assessed whether the interview extracts formed coherent patterns and whether the themes accurately reflected the dataset as a whole.

#### Phase 5: Defining and Naming Themes

Themes were defined and labeled. Through this process, the themes were refined by clarifying their core meaning. A detailed analysis of each theme and the relationships between the themes was conducted to identify potential overlaps and determine whether they were distinct from each other. The same process was conducted within and between the subthemes.

#### Phase 6: Producing the Report

Although writing and presenting the results is a continuous part of the process rather than something reserved for the final phase of analysis [44], the later stage of the work involved describing and reporting the themes, subthemes, and codes, along with selected excerpts that reflect the essence of each particular theme or subtheme. Subsequently, the themes, subthemes, codes, and chosen excerpts were translated from Swedish, the original interview language, into English.

### Research Trustworthiness

As several members of the research team have prior experience in and knowledge of ICBT, qualitative research, and qualitative research on ICBT, we reflected on any preexisting assumptions. The research team included researchers of varying ages, genders, and levels of research experience. At the time the interviews were conducted, M Baltius and EL were MSc psychology students in their final (tenth) semester. VA was a PhD student and M Berg, GA, and ML held PhDs. VA, M Berg, GA, and ML were working at Linköping University in research and teaching roles. VA, M Baltius, EL, and M Berg are women, whereas GA and ML are men. M Berg, GA, and ML have prior experience with qualitative research, both within and outside the field of clinical psychology. VA had no prior experience conducting qualitative research but had completed relevant coursework at the master’s and doctoral levels. M Baltius and EL had taken master’s-level courses in qualitative methods and were supervised by the other authors.

Before the interviews, conducted by M Baltius and EL, who were not involved in the pilot RCT, the research team discussed the importance of open-ended questions and possible follow-up questions in the semistructured interview guide, as well as the importance of remaining receptive to information that might not align with previous findings or expectations. The data analysis was conducted independently by M Baltius, EL, and VA, with additional contributions from ML. GA and M Berg oversaw and supported the entire study, from the development of the interview guide to the qualitative data analysis. All authors reviewed and provided feedback on the analysis.

### Ethical Considerations

The study involved human participants and focused on their experiences; thus, it was submitted for ethical review and approved by the Swedish National Ethics Committee (Dnr 2020-02313). Participants were sent an email containing information about the study and a description of the purpose of the interview. The email also informed participants that participation was voluntary, that the interviews would be recorded, and that their information and answers would be presented in an anonymous format. The participants were also informed that they could withdraw from the study at any time without providing a reason. The research team subsequently contacted the participants by telephone and provided the same information verbally, after which informed consent was obtained. No compensation was offered to the participants.

## Results

### Overview

Of the 23 participants who were invited to take part in the study, 16 (70%) completed the telephone interview, while 7 (30%) did not take part in the study. Of these 7 invitees, 3 (43%) could not be reached by email or telephone, while the remaining 4 (57%) declined to participate, citing time constraints or lack of interest. The participants’ mean age was 51.87 (SD 18.64; range 23-78) years. Of the 16 participants, 11 (69%) were female, and 5 (31%) were male; moreover, 9 (56%) were from the initial treatment group and 7 (44%) were from the initial control group. The participants logged in to the treatment platform 34.44 (SD 18.82; range 11-85) times on average and completed an average of 6.19 (SD 1.05; range 4-7) modules. On the basis of these interviews, data saturation was considered to have been achieved.

Four main themes were identified regarding the participants’ experiences—functions of the treatment, treatment equals work, changes experienced, and wishing for something else—with 10 related subthemes. A summary of the themes and subthemes, along with examples of codes and illustrative quotes, is presented in Table 1.

**Table 1:** Overview of the main themes and subthemes, along with examples of codes and illustrative quotes.

Main themes and subthemes		Examples of codes	Illustrative quotes
**Functions of the treatment**			
	Initiating and motivating	Treatment as motivating, therapist helped to get activated, and became activated	“I found a lot of things that...like because the weather outside; it was boring; and I was tired and so on. I detest going by myself, still I need to exercise and come outdoors and so. And then, then I used this strategy to do the opposite of what one feels like doing, yes then I do an exercise. ‘Do the opposite.’ I go out, then. So, I got dressed and went out for a walk. And that was, well I...You know! Then I laughed out loud to myself. Was it this easy?” [P6; female; 67 y]
	Perspective widening	Treatment gives hope, helps identify why feeling in a certain way, and identifies other problems	“[I]f I am stressed about something, then it is easier [now after the treatment] for me to take a step back and think about why. And see if you, for example, can solve the stress in a different way than just accept that you are stressed and do not feel good because of it...If I am having a bad day nowadays, then I can have a bad day for a while and then perhaps think that “well, then it will be a better afternoon and that yes, I let this go for a second and try again later.” While before, I hung around longer with [the thought of] the bad day, so to speak.” [P11; male; 40 y]
**Treatment equals work**			
	Experience of the treatment as demanding	Demanding aspects of the treatment, obstacles to engaging with the treatment, and treatment perceived as demanding when feeling unwell	“[The treatment was] useful but tough. It is this I mean...the hardest [parts of the treatment] was probably also the best for me.” [P6; female; 67 y]
	Going from text to action	Practiced the treatment content, using strategy from the treatment, and concrete work with strategy	“[J]ust that I can go back and read, well, this was probably a thought trap, or that it happens that I automatically can think now and wonder if this is a thought trap that tries to trick me now.” [P8; female; 54 y]
	Posttreatment engagement	Experiencing use of the treatment afterward, continuous engagement with treatment strategy, and need for repetition	“[I]f one wants to get an effect, then one has to work with this all the time, yes but actually all the time, so to say.” [P7; male; 42 y]
	Participant agency	Experience of having to be active, adapting the treatment to self, and oneself has the responsibility	“My own contribution was the largest contribution. It was completely, completely decisive for how I feel today.” [P6; female; 67 y]
**Changes experienced**			
	Changes in relation to the COVID-19 pandemic	Treatment helped with managing the COVID-19 pandemic, treatment has helped managing pandemic consequences, and other circumstances helped to handle the pandemic	“[T]here are several such, eh, things, factors that should make me feel, even worse in the summer compared to the spring, but, but it did not turn out that way...and it is surely to some extent, eh, [thanks to] this [treatment] from which I have these tools and that I have received this.” [P7; male; 42 y]
	Other changes not related to the COVID-19 pandemic	Treatment has helped not only with the pandemic but also other things, experienced the treatment as helpful, and treatment helped with issues other than the pandemic	“I usually think November, like around that time, is pretty tough normally, but I felt better in November than I usually do and maybe it also has to do with the fact that I had precisely boosted on a little, like, received some tools [from the treatment] and could hold onto these tools.” [P5; female; 45 y]
**Wishing for something else**			
	Individually tailored	Treatment too general, treatment not individually tailored enough, and wish for more variation in the treatment content	“I did not really feel that it was directly related to my specific problems. And this is what happens with such a general treatment method...There were areas around the anxiety problem, they were so general because it [the treatment] should fit many.” [P4; female; 46 y]
	Contact with the therapist	Wish for verbal contact with therapist, wish for face-to-face contact with therapist, and request for other kind of contact with therapist	“One got great response, but it would also have been nice to have had someone to talk to beyond that...at least at some time. Some kind of telephone contact.” [P3; female; 28 y]

### Main Theme: Functions of the Treatment

Two different essential functions of the treatment were identified in the data corresponding to the subthemes initiating and motivating, which reflects a shift in the participants’ level of activation regarding their mental health; and perspective widening, which highlights participants’ new reflections and insights during the treatment.

#### Subtheme: Initiating and Motivating

During the treatment, participants received modules containing reading material and practical exercises, which encouraged them to take an active role in the treatment. Allowing participants to request specific modules was an aspect of the treatment that was described as motivating and prompted active engagement in and reflection about the treatment. Even when not feeling well, just doing something about it can be a good thing. Taking control by being the one creating or structuring the day’s activity was also mentioned as motivating. As another example, a participant described structure as a helpful way of getting started with positive activities and also noted that she did not need to think much about a positive activity (so-called plus activity) before engaging with it:

One thing that was more powerful than I had understood before was this about creating simple, good...Or creating structure at least, and to create simple, good “plus activities.” [I realized] that it does not have to be a big effort, but instead that you make sure to not think so much before doing it...And just think to yourself: “Mm, today I have planned to do this when I quit work. Yes, then I will do it now,” or something like that.

P5; female; 45 y

This participant stated that it did not require a big step to initiate an activity that had been on her mind. When she planned to engage in an activity, she acted on it without further deliberation. Through different aspects of the treatment, participants began, or became motivated to perform, certain everyday activities. This included activities they had enjoyed before the pandemic but did not engage in anymore. The treatment itself could also be viewed as a substitute for activities they missed because of the pandemic:

[I]t was actually fun to get to do something with the brain and to write a little and formulate as well, I thought. Because it was also...the problem I thought was that I, that I...I have been working for a long time and been active...It hit me a little hard that I...had to quit my job.

P14; female; 75 y

This participant described the treatment as stimulating. The treatment became something else in which she could become active and involved when she had to quit her job. Overall, the treatment was described as helping the participants engage with something actively, both within the treatment itself and with other activities. However, this was not the case for all. There were also descriptions of difficulty in finding motivation or energy to engage with the treatment.

#### Subtheme: Perspective Widening

Participants reported gaining new insights and broader perspectives on mental health and emotions as well as their personal situations during the treatment. The treatment helped participants identify feelings that bothered them (eg, if it was mainly fear or worry) and provide explanations for why these feelings had emerged:

I have received help to, like, [understand] what it was with this with corona...And that my freedom to make own decisions was taken away from me. Because it was this that was my problem, that, that it, independence was taken away from me. I was not allowed to go to work, I was forbidden to do things.

P4; female; 46 y

The perspective-widening function of the treatment also involved descriptions of feeling less lonely in one’s life situation during the pandemic and in handling the COVID-19 situation:

[I]t was that I felt that I got...it [the treatment] gave me power and new thoughts and to see myself from the outside. And that, and that it was not that strange what I experienced, but it was many other [people who felt the same]...From what I understood from questions and such in the treatment, I understood that it was quite common to feel this...With the stress related to COVID.

P14; female; 75 y

The participant described gaining the perspective of observing herself from the outside and realizing that she was not alone in her experiences. To summarize, this function of the treatment involved gaining wider perspectives on which feelings were present and why they were present, as well as the realization that others also struggled with similar emotions.

### Main Theme: Treatment Equals Work

Four subthemes were derived from the interviews regarding the treatment and its effects: experience of the treatment as demanding, going from text to action, posttreatment engagement, and participant agency. These subthemes corresponded to the participants’ impressions of working with the treatment, how they engaged with the text-based material in practice, their continued efforts after the 7-week treatment period, and their own role in the treatment.

#### Subtheme: Experience of the Treatment as Demanding

Participants described the treatment as demanding in terms of both time and effort. One participant reflected on the timing of the treatment and emphasized the advantage of having more free time when engaging with it:

I did this [the treatment] when I was on vacation, so it was very good. Because, basically, it takes a lot of time and effort. Like to do these things.

P8; female; 54 y

The experience of the effort required was described as both tough and nice—sometimes simultaneously:

It can...Things that are a bit nice-pain. It is nice to work with it and eh work with it structured...And still, it is tough and hurts and I had to fight very much with certain things [components of the treatment].

P6; female; 67 y

The participant experienced the treatment work as both rewarding and painful. The interviews revealed that the workload was experienced as both moderate and overwhelming. As the treatment involved many components, it could be challenging to keep up: each week introduced a new module, making it difficult to engage in other activities.

#### Subtheme: Going From Text to Action

Descriptions of participants’ engagement with the treatment and CBT strategies were provided in the interviews; for example, this could involve using a smartphone app to register thoughts of worry that occurred during the day and later dealt with during scheduled worry time (ie, when working with the worry module). Other participants mentioned keeping the CBT strategies in mind:

[S]o instead of having a new nuisance every time, you have a name for it, and then it does not become as scary...yes, now that feeling comes again, and that I, and so you call it something...then it, like, this went good last time so it will also go well this time.

P7; male; 42 y

By noticing and recognizing a feeling, the participant practically used a common first step in emotion regulation. This involved observing the upcoming emotion in that very moment. The practical implications described in the interviews could also be on a more abstract level. A participant described how she had worked with behavioral activation:

I do not use this activity plan within the daily routine as strictly as I did then [during the treatment weeks]. But it exists in the sense that I think what I will do, eh. I engage in some of these plus activities each day.

P6; female; 67 y

Although this participant was not fully using behavioral activation as described in the treatment, the thought of “plus activities” was on her mind. In sum, the subtheme treatment equals work involved being influenced by the treatment and using it in both concrete and abstract ways.

#### Subtheme: Posttreatment Engagement

This subtheme involved statements regarding the continued use of the treatment strategies after completing the 7-week treatment schedule. The treatment could be viewed as continuously ongoing rather than something to finish:

I think that CBT and these things, they are actually, yes, it is something ongoing. It is nothing, it is not a wound that heals and then you are done, but it rather leads to new questions. You become equipped with a toolbox.

P9; female, 70 y

In the interviews, there were different ways in how engagement with the treatment was described; for instance, a participant described how he tried to handle feelings by viewing difficult feelings as a beach ball, metaphorically speaking:

[T]here are certain keywords...that I have taken with me, yes, that I still work with, so, yes, like this beach ball is one example. That if you notice that, “now I am trying to smooth things over here,” and pretend like that it does not exist and so on, eh, and I should not do that then, but I should try, you are supposed to be with your feelings, I should learn to live with this, so to speak.

P7; male; 42 y

Participants shared that repeating a specific strategy was also considered important and helpful, especially in stressful situations, when strategies were not routinely applied. It was clear that the treatment had not fully prevented the occurrence of difficult situations, feelings, and thoughts but had rather provided strategies and ways to handle problems.

#### Subtheme: Participant Agency

Participants described their own role in the treatment, including control and responsibility; for instance, engagement was described as crucial for the treatment to work: although it was individually tailored in terms of the choice of modules, it was standardized because the specific modules were the same for all who received them. This is exemplified by a participant’s description of her experience of the support she received from the therapist:

[I]t was the support I felt I needed there and then that I got because the other parts of the treatment are such things that lie with me, it is I who must like do the things, it is no one else but I who must do this and challenge.

P8; female; 54 y

The participant emphasized her own responsibility in the process, explaining that no one else—not even the therapist—could take over. Another participant gave an example of the demands she experienced:

Just this that one must go into the deep to be able to understand and absorb something. Yes. And making it to be my own [treatment], how can I use this in a good way?

P15; female; 75 y

This participant underscored the necessity of engaging in the treatment to make it relevant and adapt it to her situation. Various descriptions of the participants’ relationship with their therapist emerged in the interviews, with the idea of being a team serving as 1 example. The relationship was described as collaborative work, rather than a one-way relationship in which the therapist delivered the treatment. Along these lines, a participant emphasized being in the driver’s seat with responsibility:

[I]t is clear that it [the treatment] makes huge demands that you are the active one, you should be the proactive, you should drive it.

P10; male; 48 y

This interviewee described an experience characterized by responsibility and the obligations placed on him as a participant. Overall, the participant described his role in the treatment as vital but not as standing alone. In the interviews, it became clear that this role can only be taken on by the participants themselves to achieve the desired treatment effects.

### Main Theme: Changes Experienced

As described by participants, the changes that emerged during and after the treatment differed in both cause and content. Accordingly, 2 subthemes were identified: changes in relation to the COVID-19 pandemic and other changes not related to the COVID-19 pandemic.

#### Subtheme: Changes in Relation to the COVID-19 Pandemic

Changes regarding symptoms related to the COVID-19 pandemic were described. As explained by a participant, the treatment could be used to manage the circumstances brought about by the pandemic:

I barely meet anyone. But...eh...and then there are many who ask, how do you cope? Yes, but I think I cope because I have...all this other stuff [the treatment] that I work with, and in that way, I have been helped by these tools.

P9; female; 70 y

Another way the treatment was described as helpful was by changing how the pandemic was viewed, for instance, how much worry it caused and how it was managed. These psychological changes were not necessarily linked to how the pandemic situation changed; for example, a participant described her mental health improving despite the pandemic worsening:

It was therefore it became like...almost another life. Before CBT, I did not know exactly how I felt, I can remember that. And now after this, I mean the pandemic, this has not changed. It [things] has even become much worse now for me. But I feel much better, and I can handle it.

P6; female; 67 y

The interviews also included accounts in which participants attributed their improved mental health to factors beyond the treatment. Other changes in their everyday life were also described, such as getting a new job.

#### Subtheme: Other Changes Not Related to the COVID-19 Pandemic

The treatment was also described as having an impact on issues not directly related to the pandemic. One participant realized that the treatment material would not only be useful during the pandemic but also in other areas of life:

[A]fter I got started, I felt that this is good and will be good for me like, it is not only now during the pandemic, but also like, yes, but you know, in working life and so on.

P5; female; 45 y

The treatment was also described as addressing psychological symptoms that existed before the COVID-19 pandemic began:

I was quite stuck before I started with the treatment, so, it was nice to get some keys to how I unlock my problems that I have actually had for a long time.

P3; female; 28 y

### Main Theme: Wishing for Something Else

This main theme consisted of 2 subthemes: individually tailored and contact with the therapist. The main theme and subthemes reflect treatment components that participants wished had been included or implemented differently. Although not included in this theme or highlighted as a separate theme, participants also described the treatment as helpful and useful and the treatment format as applicable. The treatment was described as individually tailored, with the possibility to request modules relevant to one’s situation and symptoms. Therapist contact was appreciated and at times described as essential. Nonetheless, as illustrated by this theme, participants expressed wishes for additional treatment components.

#### Subtheme: Individually Tailored

The treatment was individually tailored through the selection of modules, while the content of each module remained unchanged. As a result, the material was not always fully applicable to each participant. Even if the focus of the module had been judged as potentially helpful, the treatment was also described as too general to be useful:

[T]he treatment did not give me so much as it was too general. It was not as specific as I wanted it to be. Sure I could choose, eh, which areas I wanted to look into further or wanted to work with and so. But it was still that every area was so very general and large, that it was difficult to apply specifically for what I felt.

P4; female; 46 y

This participant wanted more specific treatment material. To make the treatment more user-friendly, accessible, and helpful, participants offered some concrete suggestions; for instance, a participant mentioned both her own preferences and what she thought could help other people:

[W]hat I missed very much was that there should have been small, short video recordings...I would really like to have visual, more that...and then it can be good to listen to someone who reads the text...I think that could make it easier to absorb the text for some people.

P9; female; 70 y

#### Subtheme: Contact With the Therapist

There were some suggestions or requests for different types of contact with the therapist within the treatment; for instance, a real-time chat function as a complement to the asynchronous text message function was suggested. Alternatives to internet-based contact with the therapist were also mentioned, such as telephone contact:

[I]t would have been good if one had both, at least for me. Like some human contact too and not just email...that there could have been a mix of it email and telephone contact then I think it would have been better.

P10; male; 48 y

Another example of a request was for face-to-face meetings with the therapist. One participant expressed a wish to meet the therapist in person to discuss the problems she was experiencing:

I would personally have needed a little more direct personal contact...I felt I did not have anyone to bounce ideas directly with. Well, sure, I could write to my therapist, and I received some good answers, but I would have needed to meet someone. Where one can sit down and discuss things...I have nothing against CBT, well, I believe in the method...but I would have preferred a face-to-face CBT and not an internet-based.

P4; female; 46 y

## Discussion

### Principal Findings

This study aimed to explore participants’ experiences of undergoing individually tailored ICBT with weekly therapist support during the COVID-19 pandemic. The intention was also to investigate the experienced usefulness and possible limitations of the treatment. After semistructured telephone interviews, data analysis, and interpretation, 4 main themes and 10 underlying subthemes were identified. The 4 main themes generated were functions of the treatment, treatment equals work, changes experienced, and wishing for something else.

### Limitations

There are several limitations to this study that should be addressed. First, as in any study involving participants who have received treatment, there is a risk that social desirability influenced the responses. The interviewers had not had any contact with the participants before the interviews, that is, they were not the participants’ therapists. However, participants may still have withheld honest responses to avoid disappointing the interviewers, who are members of the research team. Hopefully, participants understood that they had the chance to express what they did not like about the treatment and suggest changes. Questions about this aspect were included in the interview guide; moreover, the people who administered the interviews were not involved in the project when the pilot RCT [22] was conducted. Second, the shortest interview lasted only 8 minutes, while the mean interview length was 19 minutes, with some interviews being longer. There is a risk in all interviews, but especially in the shortest ones, that important aspects may have been missed, and the shorter interviews could be due to the interviews being conducted over the telephone. However, it could also be that the participants experienced that it was more convenient to give feedback when not having eye contact with the interviewer. In addition, in line with the discussion by Biliunaite et al [46], participants who agreed to take part in the study could be more positive about the treatment compared to those who declined to participate. The results might have differed if the 7 invited individuals (n=3, 43% were unreachable; n=4, 57% declined) who did not participate had been included. We recognize the need for broader recruitment to further explore the experiences of individuals who felt that the ICBT was not suitable for them. Moreover, the participants were interviewed approximately 4 to 6 months after completing the treatment. There is a risk of recall bias because people in the control group, receiving the treatment later and completing the interviews closer to the treatment, may have had the treatment more readily available in their memory. However, this was not observed as a systematic problem by the interviewers or by the other authors when analyzing the data. The relatively long period of time between the treatment and the interviews could have affected what the participants remembered from the treatment, and answers could have been different if the interviews had been conducted directly after the treatment concluded. At the same time, the delay between treatment and interview could also have allowed participants time to reflect and assess what, if anything, they found useful regarding the treatment, which was part of our aim. Thus, there was an opportunity to investigate whether participants continued to use the treatment material after treatment conclusion and whether this had been helpful to them, as illustrated by the identified subtheme posttreatment engagement.

It is possible that our prior understanding and knowledge of ICBT impacted the analysis of the results. Several members of the research team have extensive experience with ICBT and with qualitative methodology. Furthermore, all authors, except for the 2 interviewers, were involved in the pilot RCT [22]. However, this is not necessarily a problem because researcher subjectivity can be viewed as an advantage [47]; for example, when participants sometimes referred to a certain module or assignment within the treatment, the interviewers and authors could follow up with clarifying questions because they were familiar with the treatment approach and the modules. It is also important to acknowledge that there could be other possible ways to code and interpret the participants’ statements. Furthermore, it is possible that we would have ended up with different results if we had used an alternative qualitative analysis method. The process of thematization or pattern identification can be found in other qualitative approaches, such as interpretative phenomenological analysis or grounded theory [45]. However, both interpretative phenomenological analysis and grounded theory are theoretically grounded, in contrast to TA [44]. TA is not inherently linked to any particular theory or epistemological perspective, which we deemed most suitable for our purpose. Moreover, the results might have differed if a different semistructured interview guide had been used, for instance, one with a greater focus on core CBT principles. Our intention with the broad questions was to provide participants with as much space as possible to freely reflect on their experiences, particularly because the ICBT program was individually tailored, and not all participants engaged with the same materials and CBT strategies. However, more focused questions could have served as a reminder of the components to which the participants had access during the treatment and potentially elicited more detailed descriptions of their experiences.

It is also worth mentioning that, as with other qualitative research, the possibility of generalizing the results is limited and is not even a goal of the research [48]. However, the themes identified do provide us with information on how the participants in this study experienced undergoing ICBT during the COVID-19 pandemic. The results are to a great extent similar to those of previous qualitative studies in the ICBT research field [49,50]. This supports the notion of transferability, which, rather than generalizability, is commonly used as a quality criterion in qualitative research [51]. As such, the findings may also be informative for other ICBT programs tested under more typical conditions, when the COVID-19 pandemic is no longer a major societal concern.

### Comparison to Previous Work

Considering the first main theme—functions of the treatment—participants described in different ways how the treatment and their work with the treatment affected them. The motivating and activating function that was described aligns with previous findings regarding increased self-efficacy as a positive aspect of ICBT [34]. The function of the treatment as perspective widening also mirrors previous qualitative studies in the ICBT field [49,50,52,53]. Participants described gaining new insights and a broader understanding of their mental health, contributing to an increased sense of control. Similar experiences have been reported by other individuals who underwent ICBT during the COVID-19 pandemic [37]. As important as these findings are, it is not surprising that the treatment can help one understand oneself and the problems experienced, in particular given that psychoeducation is part of the treatment. Psychoeducation alone has been shown to be effective for symptoms of depression and psychological distress [54]. A possible extension of this finding is that identifying and understanding one’s feelings might be therapeutic in itself. Affect labeling—putting feelings into words—has been observed to have an emotion-regulating function [55].

Regarding the second main theme, related to the treatment effects being dependent on work and effort, participants described the treatment as demanding in terms of time and energy. This has been reported before in a previous qualitative study on ICBT [50]. In this study, descriptions about this experience were salient both during the treatment period and afterward, which illustrates the importance of motivation. Moreover, participants described their role and agency in the treatment, which can be seen as reflecting not only self-efficacy but also a recognition of their responsibility for their own mental health. This active approach to the treatment, both during the treatment period and afterward, is in line with previous findings that emphasize the importance of processing and applying the treatment material [49], as well as the value of autonomy and self-sufficiency [52].

The original purpose of the pilot RCT [22] was to investigate the effects of ICBT on psychological symptoms related to the COVID-19 pandemic. It became evident in this qualitative investigation, as illustrated by the third main theme about the changes experienced, that working with the treatment was not isolated to pandemic-related symptoms. The modules used in the treatment were derived from previous ICBT studies, conducted before the pandemic, and dealt with problems that very well could have been present before the pandemic. However, the modules were adapted and focused on the COVID-19 pandemic context. In addition, this is in line with the notion that comorbidity is common [56] and the conception that common underlying mechanisms, such as anxiety sensitivity [57], contribute to a range of psychological symptoms [58,59].

The fourth and final theme concerned the participants’ wishes and suggestions regarding how the treatment format and the therapist contact could have been designed differently to better suit their needs. Some of the suggested changes have been raised in previous studies [46,50,60]; for example, the experience of ICBT not being sufficiently individually tailored has been reported both before [61] and during the COVID-19 pandemic [36]. It was mentioned that the existing form of tailoring was not sufficient to make the treatment useful for everyone. People in general and the participants in this study were impacted by the COVID-19 pandemic in very different ways, with some, for example, experiencing worry as their main problem, while others reported feeling more depressed. Creating different combinations of modules in this study was an attempt to address this variety of needs, but the way the tailoring was implemented could be improved further in future studies [62].

Similar to the findings by Asplund et al [50], participants in this study expressed a need for extended or alternative forms of support and contact with their therapist, such as through telephone or video. At the same time, other participants indicated that they did not feel a significant need for extended contact with their therapist or appreciated that the contact was only through asynchronous text messages. No participants reported problems with the technology, such as logging into the platform. However, as discussed by Patel et al [35], this may be due to the likelihood that the participants interviewed, who had chosen to take part in an ICBT study, were computer literate.

The findings of this study are valuable in enhancing the understanding of how participants may experience undergoing ICBT, particularly in the context of the COVID-19 pandemic. The results largely align with previous research on experiences of internet-based interventions, highlighting themes such as the development of understanding and perspectives on thoughts and feelings [37], the importance of the participant’s active involvement in the treatment process, and the significance of both treatment personalization and individual feedback [35]. These insights further expand the current perspectives on how ICBT may be perceived, elucidating the functions the treatment is seen to serve, how participants’ engagement with the treatment is described and experienced, and what aspects would need to be altered or improved. A distinctive contribution of this study, in comparison to previous research conducted both before [35] and during the COVID-19 pandemic [36,37], is the exploration of participants’ perceptions of the underlying causes and specific content of the changes attributed to ICBT. This includes how participants described changes in their mental well-being, both in relation to the COVID-19 pandemic and in more general terms.

### Future Research

To gain further insights into how people experience undergoing ICBT focused on psychological symptoms related to the COVID-19 pandemic and to explore the experiences of undergoing ICBT in general, it would be valuable to interview an even broader range of participants. This includes all participants, from those completing a single module to those who completed all modules and remained active throughout the treatment. Demonstrating the effectiveness of a therapy does not necessarily yield evidence regarding why the therapy works [63]. Qualitatively investigating what participants perceive as helpful or less helpful could be a way to gain more insight into their experiences, including reasons for ending treatment or dropping out prematurely. In addition to exploring the experiences of a broader sample of participants, it would be necessary to investigate participants’ experiences throughout treatment. This would allow adjustments to be made during the treatment process, rather than insights emerging only after treatment ends. Furthermore, comparing experiences during and after treatment could help evaluate whether any adjustments could improve treatment outcomes and adherence.

It would be relevant to implement changes and further develop the material of this ICBT program based on the findings of this study. This would enable a comparison of treatment outcomes between the revised and original program versions. At the same time, several aspects of the program were described as suitable and helpful. Other ICBT programs, not necessarily focused on psychological symptoms during the COVID-19 pandemic, could also be developed based on these results because a considerable portion of what participants described in the interviews pertains to generic aspects of ICBT. Furthermore, it could be valuable to mix quantitative and qualitative methods to evaluate ICBT.

As ICBT relies on technology, including treatment platforms that feature exercises and communication with a therapist, it is essential to examine participants’ experiences with the technology itself. This should include an exploration of how comfortable participants feel with the technology based on their technical competence and sense of security, as well as how the technology may influence their overall experience of the treatment.

Finally, based on the subtheme contact with the therapist, it would be of interest to investigate experiences of ICBT in which participants can choose their preferred mode of therapist contact. Gaining further understanding of participants’ preferences for both treatment content and therapist support could inform further development of ICBT.

Overall, this study should be followed by other similar investigations into how ICBT programs are experienced. Each new treatment program and controlled trial could be complemented by a qualitative study because such studies provide additional information and can be useful when refining an intervention [64].

### Conclusions

This study investigated participants’ experiences of undergoing ICBT during the COVID-19 pandemic in the summer of 2020, focusing on psychological symptoms related to the pandemic. The results largely align with previous research in the field, but pandemic-specific findings were also identified. The treatment seemed to serve various functions, such as helping to widen participants’ understanding of their own feelings; it required effort both during and after the treatment period; and its format, in terms of the level of individual tailoring and therapist contact, did not suit everyone’s needs. Moreover, the treatment content seemed to be relevant not only for psychological symptoms related to the COVID-19 pandemic but also for other symptoms. In other words, participants described changes in their well-being as a result of the treatment in ways that extended beyond the pandemic context, highlighting the potential of ICBT to facilitate psychological improvements independent of situational factors.

## Appendix

Multimedia Appendix 1 Interview guide.

## Abbreviations

CBT: cognitive behavioral therapy
COREQ: Consolidated Criteria for Reporting Qualitative Research
ICBT: internet-based cognitive behavioral therapy
RCT: randomized controlled trial
TA: thematic analysis

## Data Availability

The data set generated or analyzed during this study are not publicly available due to the inclusion of sensitive information but additional examples of quotes for each theme are available from the corresponding author on reasonable request.
