# Supplementary material for: Experiences of Individually Tailored Internet-Based Cognitive Behavioral Therapy During the COVID-19 Pandemic: Qualitative Interview Study
Source: JMIR Form Res. 2025 Jun 11;9:e66908. doi: 10.2196/66908 (PMC12204044; doi:10.2196/66908)
Supplement: Multimedia Appendix 1 [file formative_v9i1e66908_app1.docx]

**Appendix**

**Appendix 1**

The semistructured interview guide followed when conducting the interviews with the participants.

Hi!

My name is (one of the interviewers’ names), and I am part of the CoronaCope-research group. Thank you for participating in an interview about your experience of the ICBT program four to six months ago. The ICBT aimed to address psychological symptoms related to the pandemic. Participation of this interview is voluntary, and you can cancel at any time. Your answers will only be used in research purpose and nothing else. The interview will be recorded. Do you have any questions? (Time for discussing questions and orally confirming consent before continuing the interview.)

I will start the recording now.

Questions:

- What was your expectations of the ICBT treatment before it started?
- How did you experience the treatment? (Feelings, thoughts, reflections? Probing questions about feelings and thoughts, e.g. could you elaborate?)
- How did you experience the contact with the therapist?
- What aspects do you think contributed when deciding to continue or to quit working with the treatment?
- How has the treatment affected you or your situation? (Do you experience the treatment as helpful or not? In what way(s)?)
- Was there anything challenging or less useful with the treatment?
- Is there anything that you use from the treatment today? If so, what?
- What are your feelings or thoughts of your own effort within the treatment?

That was the last question of this interview. Do you have any questions or other reflections before we end? Thank you so much for your answers and your time!
